# Anaerobic and Aerobic Energy System Contribution During Maximal Exercise: A Systematic Review

**DOI:** 10.1007/s40279-026-02414-7

**Published:** 2026-04-11

**Authors:** Paul B. Gastin, Haresh T. Suppiah

**Affiliations:** Sport, Performance, and Nutrition Research Group, School of Allied Health, Human Services and Sport, La Trobe University, Melbourne, VIC 3086 Australia

## Abstract

**Background:**

The capacity to generate energy during exercise is dependent on the bioenergetic pathways within muscle cells. These pathways include the phosphagen, glycolytic, and oxidative phosphorylation systems, which work together to resynthesize adenosine triphosphate (ATP) and meet the energy demands of exercise. Understanding the relative contributions of these systems during maximal exercise has theoretical and practical importance for optimizing athletic performance and training.

**Objective:**

This study aimed to systematically review literature on the relative contributions of anaerobic and aerobic energy systems during single bouts of maximal exercise across various durations. The goal was to consolidate evidence from multiple methodologies to provide a more precise understanding of energy system interactions and inform both theoretical knowledge and practical applications in sports performance and training.

**Methods:**

A systematic search was conducted across seven electronic databases, including CINAHL, Cochrane, Embase, MEDLINE/PubMed, Scopus, SPORTDiscus, and Web of Science, covering studies from 1984 to January 2020. Eligible studies included peer-reviewed, English-language research that examined energy system contributions during maximal exercise in adults (≥ 18 years). Data extraction focused on participant details, exercise trial specifics, assessment methods, and relative energy system contributions. Studies were categorized on the basis of their assessment approach. Nonlinear regression modeling was used to estimate anaerobic and aerobic contributions across different exercise durations.

**Results:**

A total of 102 studies were included, providing 311 individual data points (mean results from studies comprising 78% male, 12% female, 11% mixed adult samples). The oxygen deficit (OD) method was used in 66 studies, mixed methods (MM) in 33 studies, and theoretical models (TM) in 7 studies. The data reviewed indicated that the anaerobic system predominates in short-duration maximal exercise, up until approximately 75–80 s. The maximal exercise duration that derived equal contributions from both the anaerobic and aerobic energy systems was 78.6 s (95% confidence interval [95% CI] ± 1.1 s), with longer durations contributing an increasingly greater proportion of aerobic energy to the total energy supply. Comparison of regression curves showed no differences between running and cycling or training status but revealed small significant effects of measurement method and pacing strategy.

**Conclusions:**

This review refines previous estimates of energy system contributions during maximal exercise, reaffirming the dynamic interplay between anaerobic and aerobic metabolism. The findings emphasize the importance of considering both energy systems in training strategies and highlight the need for more precise measurement techniques. Athletes and coaches can optimize performance by tailoring high-intensity training, work–rest ratios, and pacing strategies to improve anaerobic capacity, oxygen uptake efficiency, and short-term recovery.

**Supplementary Information:**

The online version contains supplementary material available at 10.1007/s40279-026-02414-7.

## Key Points

**Table:** 

The body relies on three integrated bioenergetic systems—the phosphagen, glycolytic, and oxidative phosphorylation pathways—to regenerate ATP during exercise. Their relative contributions depend on exercise intensity and duration, with anaerobic metabolism predominating in short maximal efforts and aerobic metabolism becoming the primary supplier during longer efforts. The crossover between anaerobic and aerobic predominance occurs at approximately 75–80 s of maximal exercise.
Quantifying anaerobic energy release during whole-body maximal exercise remains challenging. As direct invasive measures are impractical, researchers rely on indirect approaches such as oxygen deficit, mixed metabolite-based methods, and theoretical models. Each approach incorporates assumptions and methodological variations that contribute to inconsistencies in reported values.
Oxygen uptake increases more rapidly during high-intensity exercise than traditionally appreciated, making a meaningful aerobic contribution even in brief maximal efforts. Faster oxygen kinetics can enhance speed-endurance, delay fatigue, and improve performance, particularly when supported by effective pacing strategies.
Athletes, coaches, and practitioners can optimize performance by tailoring training intensity, work–rest ratios, and pacing strategies to the specific energy system demands of their event. Appropriately prescribed high-intensity training can enhance both anaerobic and aerobic pathways, supporting performance across a wide range of sports.

## Introduction

The capacity to generate energy, or produce work, during exercise is dependent on the bioenergetic pathways within the muscle cell. The energy for muscle contraction comes from the hydrolysis of adenosine triphosphate (ATP). As ATP exists in very low concentration in the muscle and regulatory mechanisms prevent its complete degradation [1, 2], the body has evolved well-regulated pathways to regenerate ATP and enable muscle contraction to continue [3]. The three bioenergetic pathways involved in the resynthesis of ATP during exercise are the phosphagen, glycolytic, and oxidative phosphorylation energy systems (for comprehensive overviews refer to [4–10]). Anaerobic (without oxygen) metabolism, which comprises substrate-level phosphorylation via the breakdown of phosphocreatine (PCr) and muscle glycogen (the phosphagen and glycolytic systems, respectively), is ideally suited to provide immediate and short-term energy delivery during explosive and high-intensity efforts. The anaerobic pathways are capable of regenerating ATP at high rates but are limited by their capacity, primarily a result of PCr and muscle glycogen depletion, the accumulation of metabolic byproducts, increased intramuscular acidity, and strong ion fluxes during exercise [6, 11]. Aerobic metabolism, however, represents the oxidative phosphorylation of carbohydrates and fats in the mitochondria, requires oxygen, and provides energy over the long term. The aerobic energy system has an enormous capacity to produce ATP, yet its power is limited by the rate of oxidative phosphorylation and the respiratory and cardiovascular systems’ ability to deliver oxygen to the working muscles [12, 13]. Investigating the interplay of the energy systems provides critical insights for both theoretical knowledge and practical applications in athletic training and rehabilitation.

As a function of their differing characteristics and contrasting powers and capacities [6, 8], the three energy systems operate in an integrated and seamlessly efficient manner to replenish ATP and meet the high, often sustained, and diverse energy demands placed on the body during exercise [14, 15]. No single system is responsible for the energy delivery for gross motor activity beyond a few contractions, as energy provision relies on simultaneous participation of all three pathways, although a predominant energy system will emerge on the basis of the nature of the activity or sporting event. This predominance has historically, although misleadingly, resulted in activities being labeled on the basis of their physiology (e.g., anaerobic or aerobic) rather than their activity (e.g., explosive, high intensity, endurance-intensive) [16]. Ergometric assessment of the power and capacity of the energy systems is theoretically and practically appealing [17, 18], although the engagement and contribution of each of the energy delivery pathways in maximal efforts renders the approach unsuitable beyond providing a power or work output associated with a specific duration of maximal exercise (e.g., 10 s, 30 s, 90 s). While measures of peak power, time to peak power, total work, and time to exhaustion provide meaningful athlete profile and performance data, they cannot be used to quantify energy release and the labeling of these tests (e.g., alactic work capacity, anaerobic work capacity) as energy equivalents or proxies can be misleading. One exception might be found in the concept of critical power (CP), originally proposed by Monod and Scherrer [19] in 1965 to describe the highest exercise intensity that can theoretically be sustained for an extended duration without leading to fatigue or exhaustion. CP is derived from the relationship between power output and time to exhaustion (from multiple maximal trials), forming a hyperbolic curve where the asymptote represents CP [20–23]. While CP is an important endurance metric [20, 24], the curvature constant (*W′*) in the hyperbolic model (or *Y*-intercept in the linear model) is thought to represent the finite amount of work that can be done above CP and has therefore been considered a measure of anaerobic work capacity [19, 23, 25–27].

The quantitative contribution of each energy system to maximal exercise has been a topic of considerable interest in exercise physiology for more than a century, with early research focusing on the measurement of oxygen uptake during and after exercise [28, 29]. These early studies led to the development of the concept of oxygen deficit (OD), which represents the difference between the oxygen required for a given exercise intensity and the actual oxygen consumed [29–31]. Oxygen deficit provides an indirect measure of anaerobic metabolism during exercise, and despite the limitations of and contrasting views on the OD method (see [32]), it remains one of the most widely used for estimating the relative contributions of the anaerobic and aerobic energy systems during maximal exercise [33]. The OD method requires the measurement of oxygen uptake during a series of submaximal exercise bouts to establish a linear relationship between power output or speed and oxygen uptake. This relationship is then extrapolated to supramaximal exercise intensities to predict the theoretical oxygen demand or cost corresponding to the workload being completed [31]. The accumulated OD is then calculated as the difference between the predicted oxygen demand and the actual oxygen consumed during the exercise bout. The OD method is a two-component model that attempts to quantify anaerobic and aerobic energy release in oxygen (O_2_) equivalents.

In addition to the OD method, several other methods have been used to estimate anaerobic energy release and the relative contributions of the energy systems during maximal exercise. These include the measurement of muscle and blood metabolites (e.g., ATP, PCr, lactate, pyruvate) before and after exercise [34], the use of theoretical mathematical models based on data in literature [35–38], and the measurement of the fast component of the excess post-exercise oxygen consumption (EPOC) [39–41]. These methods represent a three-component model whereby the anaerobic energy release is broken down into ATP–PCr (i.e., phosphagen) and glycolytic (i.e., nonoxidative glycolysis) components. Irrespective of the approach used, the quantification of anaerobic release during exercise is challenging [42], as direct measures utilized during small muscle group exercise cannot be extrapolated to whole-body exercise [43, 44] and the invasiveness of biopsy and arterial–venous measures is unsuitable for athletic assessment. As such, there is no universally accepted method for the assessment of anaerobic energy release in whole-body exercise [15], and all indirect, minimally invasive methods seem reliant on some form of estimate, assumption, or limitation (for examples see [31, 32, 34, 37, 38, 45–47]).

**Fig. 1:**
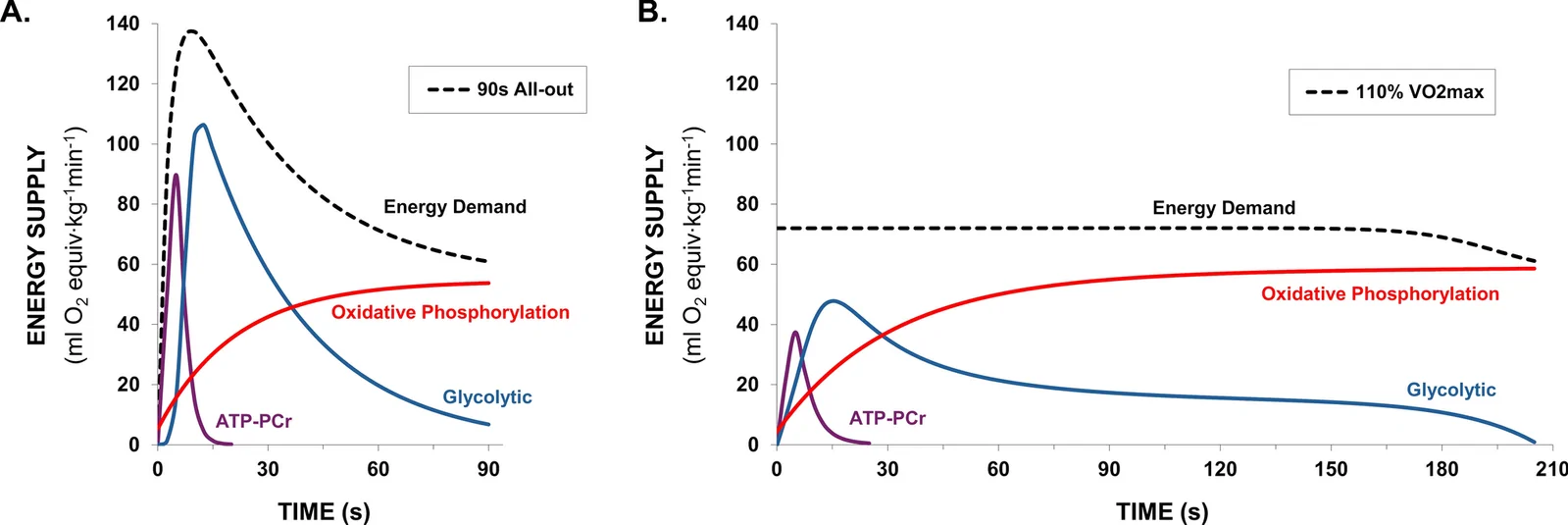
Relative contribution of the three energy systems to the total energy supply during (**A**) 90 s of all-out cycle exercise and (**B**) exhaustive constant intensity cycle exercise at 110% $\dot{V}$O_2_max. The participant was a male endurance-trained triathlete ($\dot{V}$O_2_max = 64.9 mL/kg/min; 90 s all-out oxygen deficit = 65.8 mL/kg; 110% $\dot{V}$O_2_max oxygen deficit = 69.2 mL/kg). At the conclusion of exhaustive maximal exercise at these intensities and durations, the anaerobic capacity (ATP–PCr + glycolytic) is fully spent, with the final power output (i.e., energy demand) of exercise approximately equivalent to the energy supplied by oxidative phosphorylation. $\dot{V}$*O*_*2*_*max* maximal oxygen uptake, *ATP* adenosine triphosphate, *PCr* phosphocreatine. Data from Gastin et al. [90]. Adapted from Gastin [14], with permission. Figure available as an animated GIF (Online Resource 1a and 1b)

**Fig. 2:**
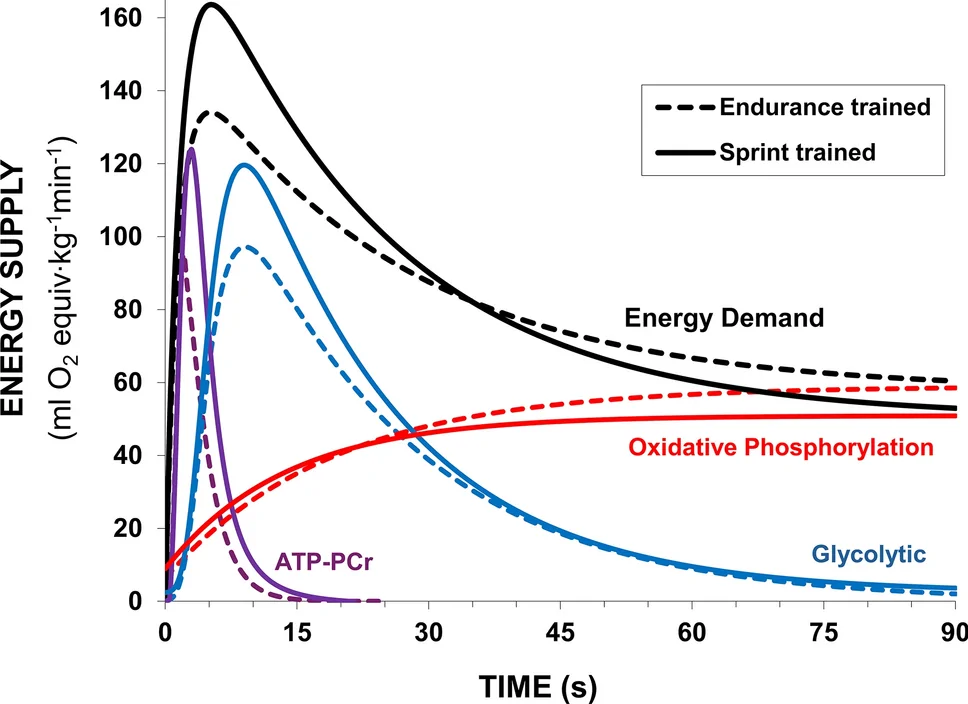
Relative contribution of the three energy systems to the total energy supply during 90 s of all-out cycle exercise comparing sprint- and endurance-trained athletes. Participants were six sprint-trained cyclists ($\dot{V}$O_2_max = 58 mL/kg/min) and eight endurance-trained triathletes ($\dot{V}$O_2_max = 65 mL/kg/min). Data from Gastin and Lawson [56]. Adapted from Gastin [14], with permission. Figure available as an animated GIF (Online Resource 2)

The balance between anaerobic and aerobic energy contribution depends heavily on the intensity and duration of the exercise [14, 45, 48–55], although other factors such as training status [56, 57], the bout of exercise [58–61], the mode of exercise [62–64], preceding exercise [65–67], diet and supplementation [40, 62, 68–70], environmental conditions [71, 72], age [73], and sex [45, 74, 75] may have some influence. Visual representation of the timing and sequencing of energy release during maximal exercise has often been used to illustrate the integration and relative contributions of the energy systems (e.g., [6, 14, 61, 76]; Figs. 1, 2). During maximal exercise lasting less than 10 s, the phosphagen (ATP–PCr) and then the glycolytic systems are the primary energy source, providing up to 95% of the total energy supply. As exercise duration increases, the contribution of the phosphagen system decreases, the glycolytic system increases then decreases, and oxidative phosphorylation increases. During maximal exercise lasting between 30 and 60 s, the phosphagen and glycolytic systems provide the majority of the energy, with anaerobic contributions of approximately 55–75% [14]. As exercise duration extends beyond 60 s, the contribution of oxidative phosphorylation progressively increases, becoming the predominant energy source during maximal exercise lasting longer than 75–90 s [14].

Despite the large body of research on the relative contributions of the energy systems during maximal exercise, inconsistencies in reported values persist, reflecting variations in methodological approaches, exercise protocols, and participant characteristics. A narrative review [14], published almost a quarter of a century ago, provided foundational insights into energy system dynamics during exercise. However, a systematic re-evaluation incorporating updated datasets and some new methods is warranted to refine these estimates. Consequently, this systematic review synthesized data from literature to clarify the relative contributions of the aerobic and anaerobic systems during single bouts of maximal exercise across varying durations. By consolidating evidence, this work outlines the methods, estimates, and assumptions that have been used to quantify energy release during maximal exercise, identify factors influencing energy system interplay, and summarize the relative energy system contribution to varying maximal exercise durations.

## Methods

### Search Strategy

The objective of this study was to systematically review published values of the relative contribution of the anaerobic and aerobic energy systems during single bouts of maximal exercise. A systematic search of seven electronic databases (CINAHL, Cochrane, Embase, MEDLINE/Pubmed, Scopus, SPORTDiscus, Web of Science) from 1984 to January 2020 was performed; keywords relating to energy system interaction and relative contribution during maximal exercise were used. A research librarian assisted with developing the search strategy. The full search strategy is presented in Table 1. Database-specific subject headings were used as appropriate. In addition, a gray literature search was performed by screening Google Scholar. Previously identified eligible full texts from the original review article [14] were also included. Identified studies were exported to EndNote (Clarivate Analytics, Philadelphia, Pennsylvania, USA, Version 21, 2023) and duplicates were removed. The remaining studies were exported to Covidence systematic review software (Veritas Health Innovation, Melbourne, Australia; www.covidence.org) for title and abstract review to determine their alignment with the inclusion criteria. The full texts of articles deemed potentially eligible were subsequently examined independently by both reviewers for confirmation.

**Table 1:** Search strategy

Set	Search strategy
#1	(("percent" or "per cent") adj2 ("aerobic" or "anaerobic"))
#2	("Energy-system contribut*" or "Energy system contribut*")
#3	("Energy contribut*" or "Energy demand*")
#4	"Anaerob* system*"
#5	"Aerob* system*"
#6	("oxygen contribut*" or "O_2_ contribut*")
#7	("O_2_ deficit* " or "oxygen deficit")
#8	("O_2_ demand*" or "O_2_ contribution*")
#9	("oxygen demand" or "oxygen contribution")
#10	("accumul* oxygen*" or "accumul* oxygen* deficit*")
#11	("accumul* O_2_" or "accumul* O_2_ deficit*")
#12	("high-intensity" adj4 ("sport" or "athlete" or "exercise" or "movement*"))
#13	(Wingate adj2 test)
#14	("All-out run*" or "All-out sprint*" or "All-out swim*" or "All-out row*")
#15	("All-out ski*" or "All-out kayak*" or "All-out skate*" or "All-out exercise")
#16	("Maximal sprint*" or "maximal run*" or "maximal swim*" or "maximal row*"
#17	("Maximal ski*" or "maximal kayak*" or "maximal skate*" or "maximal exercise*")
#18	"exhausti* exercise"
#18	(Boxing or Wrestling or Skate or skiing or Swimming or Bicycling or Cyclists or rowers or kayaking or Runners or Skating)
#20	("competit* swimmer*" or "countr* skier*" or "countr* skiing" or "crawl* swim*" or "distanc* runner*" or "elit* athlet*" or "elit* swimmer*" or "endur* athlet*" or "endur* exercis*" or "endur* perform*" or "femal* athlet*" or "femal* swimmer*" or "freestyl* event*" or "front* crawl*" or "health* subject*" or "intern* level*" or "middl* distanc*" or "nation* level*" or "recreat* athlet*" or "recreat* activ*" or "recreat* train*" or "roller* skiing" or "sprint* cross*" or "sprint* cycl*" or "taekwondo* athlet*" or "train* athlet*" or "train* cyclist*" or "train* swimmer*" or "World-class athlet*" or "brake* cycl* ergomet*" or "cycl* ergomet*" or "cycl* sprint*" or "cycl* exercis*" or "cycl* perform*" or "cycl* power*" or "grade* exercis*" or "maxim* cycl*" or "maxim* effort*" or "maxim* increment*" or "maxim* intens*" or "maxim* oxygen*" or "maxim* power*" or "maxim* speed*" or "rowing ergomet*")
#21	1 or 2 or 3 or 4 or 5 or 6 or 7 or 8 or 9 or 10 or 11
#22	12 or 13 or 14 or 15 or 16 or 17 or 18 or 19 or 20
#23	21 and 22
#24	exp animals/ not humans
#25	23 not 24

### Inclusion and Exclusion Criteria

English-language, peer-reviewed human male, female, and mixed sample studies were included within this review. Studies were included if they (1) examined individuals of the age of 18 years and above, (2) used assessment protocols to determine the aerobic and/or anaerobic energy release during a single bout of maximal exercise, and (3) reported energy contribution and exercise duration data. If interventions were used, only data from the control/baseline conditions were included.

We excluded review papers, case reports, special communications, letters to the editor, invited commentaries, conference papers, and theses. Studies that measured energy system interaction and relative contribution during intermittent exercise and repeated intervals, intermittent team sport, or involved sub-maximal exercise bouts were excluded.

### Extraction of Data

The following information and data were extracted from the included studies: (1) participant and sample details (age, sex, training type, training status, sample size); (2) exercise trial details (exercise mode, duration, pace, intensity, setting); (3) method of assessment; and (4) relative energy system contribution (aerobic and anaerobic).

### Study Selection

**Fig. 3:**
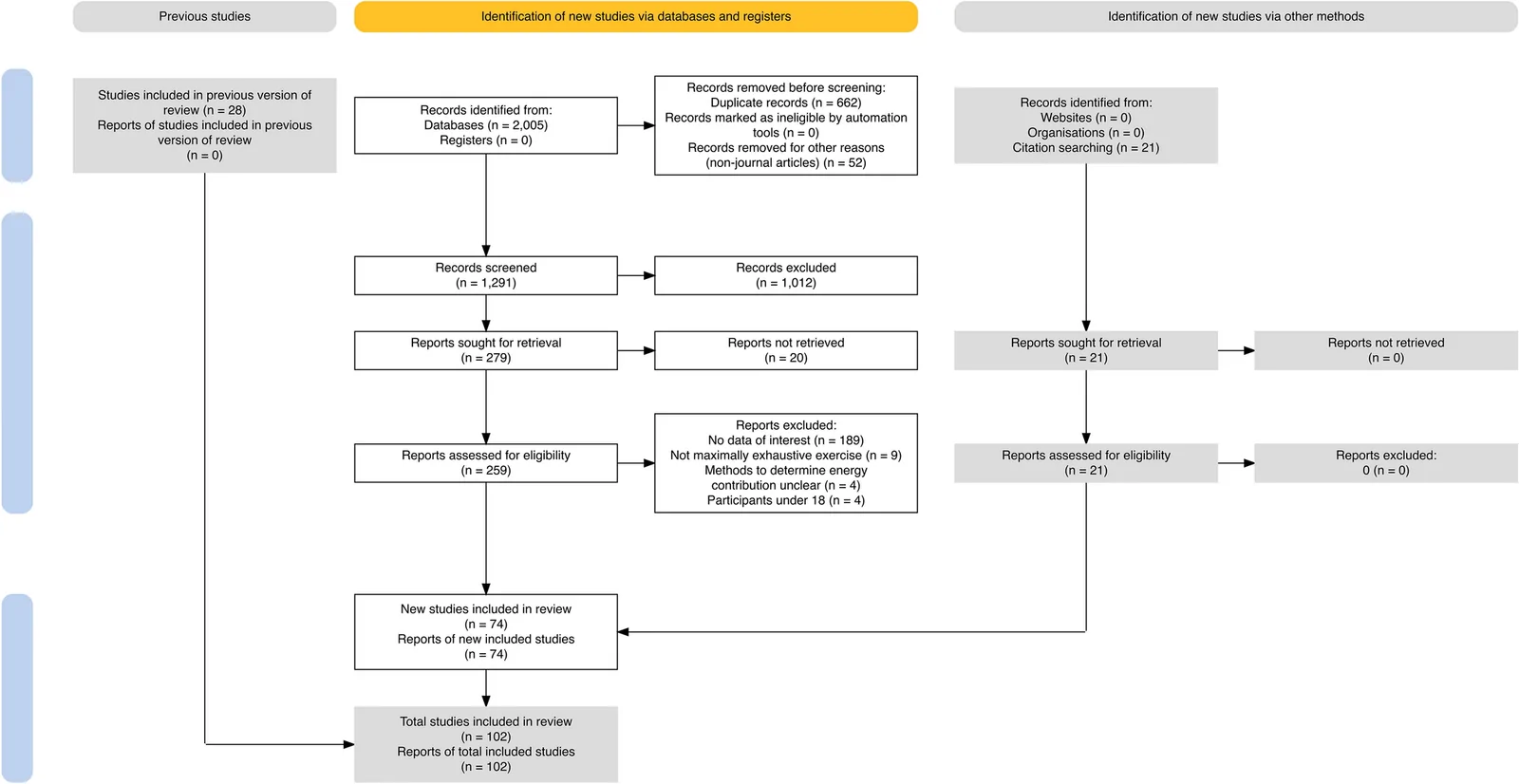
Flow of study search and selection process

The electronic database search identified 2005 records, supplemented by 21 additional records from citation searching, for a total of 2026 articles. After removing 662 duplicates, 1291 records were screened by title and abstract, resulting in 279 potentially relevant studies. Of these, 20 reports could not be retrieved and 206 were excluded following full-text screening. With the 28 eligible studies carried forward from the original review, the final number of studies included in the present review is 102. Figure 3 shows the Preferred Reporting Items for Systematic Reviews and Meta-Analyses (PRISMA) flow diagram showing the study selection process for the systematic review.

### Classification of Studies

The included studies were grouped and classified on the basis fo the approach and methods used to evaluate energy release and relative energy system contribution. Two broad groups of experimental data existed relating either to the OD method or various mixed methods that relied on direct measures or estimates of muscle and blood metabolites and/or the fast component of EPOC to estimate energy release. A third group of studies comprised theoretical mathematical modeling based on data in the literature. The category labels chosen were: (1) oxygen deficit (OD); (2) mixed methods (MM); and (3) theoretical models (TM).

### Methods Used to Calculate Energy Release and Energy System Contribution

The measurement of oxygen uptake and quantification of aerobic energy release during exercise is well established [77–79] and as such represented a consistent theoretical approach across the studies included in this review. There is a direct relationship between the oxygen uptake measured at the mouth and the whole-body aerobic production of ATP [42]. Expired gas analysis is used to calculate the energy release from the oxidation of substrates, adjusted for the nonprotein respiratory exchange ratio (RER) and the proportion of carbohydrates and fats being metabolized [80]. During maximal high intensity exercise of short duration, carbohydrates are the predominant or exclusive substrate, with the energy equivalent of oxygen typically being 20.9 kJ per liter of O_2_ in the studies reported in this review [41, 63, 71, 81–87], although 21.0 kJ [66] and 21.1 kJ [39, 64, 88] have also been reported.

The methods used to calculate anaerobic energy release and the contribution to the total energy supply are less well established and are typically indirect and based on various estimates and assumptions. Various research groups have estimated anaerobic energy release and capacity using several different methods [14, 15, 33, 89], which are defined and labeled for the purposes of this review. Irrespective of the method used, anaerobic capacity may be defined as the maximal amount of ATP resynthesized by anaerobic metabolism during a single bout of exhaustive maximal exercise [31]. Being a finite capacity, a sufficient duration of maximal exercise (~ 2–3 min [26, 31, 90]) is required to exhaust the anaerobic capacity. Many trials reported in this review are not long enough to achieve this outcome, although the exercise intensity did elicit a maximal rate of energy release during the exercise. Measurement of the relative energy system contribution for the trial was therefore still possible and of interest to this review.

### Oxygen Deficit

The term oxygen deficit (OD) was first introduced by Krogh and Lindhard in 1920 to describe the difference between the amount of oxygen required (i.e., the oxygen equivalent of the total energy demand of the work) for a specific activity and the amount of oxygen consumed during that activity. It represents the shortfall in oxygen supply, typically during the initial stages of exercise before a steady state of aerobic metabolism is achieved or during supramaximal exercise intensities that exceed the limits of aerobic energy supply. This deficit is compensated for by anaerobic metabolism, namely, the hydrolysis of PCr stored in the muscle and the nonoxidative glycolytic breakdown of carbohydrate. Early efforts to quantify the OD during maximal exercise [1, 2, 30] led Medbø et al. [31] to propose that the maximal accumulated OD (MAOD) was a quantitative measure of the anaerobic capacity.

For the purposes of this review, the term accumulated oxygen deficit (AOD) is used as opposed to MAOD, as it is inclusive of short duration maximal efforts that may not fully exhaust the anaerobic capacity and those long enough that do so. The underlying method of measuring and accumulating the difference in predicted and actual oxygen supply during epochs of time is consistent regardless of the duration of maximal exercise. The OD method is a two-component model that attempts to quantify anaerobic and aerobic energy release in oxygen equivalents.

The OD method requires the prior completion of a series of submaximal exercise efforts to establish an individual relationship between power output or speed and oxygen uptake. A presumed linear relationship is extrapolated to supramaximal exercise intensities (i.e., greater than $\dot{V}$O_2_max) to predict the theoretical oxygen demand or cost corresponding to the workload being completed. This is then accumulated across the duration of the exercise bout to quantify the OD associated with the overall exercise effort.

The protocol published by Medbø et al. [31] in 1988 has provided the foundation for most of the AOD research since. The extensive and detailed original methods have been modified and adapted to improve practicality and/or address possible theoretical concerns; however, in doing so, they may have undermined the original rigor of the proposed method. Consequently, the studies labeled under the category of “OD” in this review [2, 48–56, 60, 62, 65, 67–69, 72, 90–138] report variations in the methods used, including the number and length of submaximal exercise bouts, the *Y*-intercept used to establish the linear power output/speed–$\dot{V}$O_2_ relationship, the pacing strategy used (constant, all-out, or variable), and the intensity and duration of maximal exercise. In a comprehensive review, Noordhof et al. [33] concluded that the MAOD method, despite its limitations and inconsistencies in application, is theoretically sound, the most widely used, and probably the best noninvasive method to quantify anaerobic energy release and determine anaerobic capacity.

Within the OD category, and in contrast to the establishment of an individual power output–$\dot{V}$O_2_ relationship to determine a theoretical oxygen cost of a maximal trial, there are a small number of studies that estimated gross mechanical efficiency from literature [108, 113], or calculated it from the power output, oxygen uptake, and RER during a submaximal effort [95, 110, 134] or at the end of a $\dot{V}$O_2_max test [53, 108]. The oxygen cost of the maximal trial was then calculated from the product of gross mechanical efficiency and power output.

### Mixed Methods

Several alternative combined methods, labeled in this review under the category of “mixed methods” (MM), have been used to assess the metabolic profile of maximal intensity exercise bouts and quantify the relative energy system contribution of different energetic sources. These methods represent a three-component model whereby the anaerobic energy release is split into phosphagen and glycolytic components. The terms “alactic” (lactic acid is not formed) and “lactic” are frequently used in literature to describe these two anaerobic pathways, although more contemporary terminology refers to the pathway directly. These anaerobic components have been estimated on the basis of values in literature, estimated using indirect methods, estimated from direct measures, or estimated using a combination of these approaches. Depending on the methods and preferences of the authors, estimates of energy release of the studies labeled MM are reported as either O_2_ equivalents [45, 49–51, 69, 139, 140] or energy release in kJ [39, 47, 63, 64, 66, 71, 81–83, 85, 87, 88, 141–145].

Only one study used a direct approach by taking muscle biopsies pre and post trial to quantify phosphagen and glycolytic anaerobic energy release during maximal exercise [34]. Anaerobic ATP turnover was calculated from the values of ATP, PCr, lactate, and pyruvate before and immediately after each sprint [34]. Muscle mass was reported as 20%, with the biopsy from the vastus lateralis taken as representative of the working muscle mass.

#### Aerobic Contribution

All studies labeled as MM in this review estimated the aerobic contribution to the maximal exercise trial from the $\dot{V}$O_2_ during exercise, above a resting baseline, and converted this to an energy equivalent of O_2_ (20.9–21.1 kJ/L; see Sect. 2.6). This approach is similar to the OD method in that the area under the $\dot{V}$O_2_ curve is integrated over time and baseline $\dot{V}$O_2_ subtracted. The procedure for measuring baseline $\dot{V}$O_2_ is not always described in the studies, but has typically been individually measured at rest prior to the commencement of exercise. On at least one occasion, baseline $\dot{V}$O_2_ has been estimated from values in the literature (e.g., 3.5 mL/kg [81]).

Most studies have either ignored or did not estimate O_2_ stores in the muscle when calculating the aerobic contribution to exercise. Although small in magnitude, this is an unfortunate oversight. For those that did include O_2_ stores in their calculations, 2.3 mL O_2_/kg [45] and 6.0 mL O_2_/kg [46] have been reported.

#### Phosphagen Anaerobic Contribution: Excess Post-Exercise Oxygen Consumption

Approximately half of the studies labeled as MM in this review used the fast component of the excess post-exercise oxygen consumption (EPOC) to estimate the phosphagen (i.e., ATP–PCr) energy contribution [39–41, 63, 64, 66, 69–71, 81, 84, 85, 88, 140, 144, 146, 147]. This method assumes that the $\dot{V}$O_2_ consumed in the early phases of recovery from maximal exercise is used to reconstitute the high energy phosphates used during exercise. The fast component $\dot{V}$O_2_ has been shown to closely relate to the amount of oxygen required to resynthesize PCr immediately post exercise (*r* = 0.89) [148]. The time course of the oxygen uptake in the recovery after exercise is typically interpolated using a bi-exponential function (Fig. 1 in Beneke et al. [39]) and used to estimate the anaerobic phosphagen energy release. Integration of the exponential is calculated from the amplitude and time constant of the fast component (see Eqs. 2 and 3 [64]) with the “alactic” energy release estimated (Eq. 3 [64]) using the caloric equivalent of O_2_ [149, 150]. The caloric equivalent used for this conversion has differed slightly between authors with 20.9 kJ per liter of O_2_ [41, 63, 71, 81, 84, 85], 21.0 kJ [66], or 21.1 kJ [39, 64, 88] all being reported. Most authors have used a bi-exponential model based on the work of Beneke et al. [39], and while some have assessed and confirmed it as preferable [64], others have found no difference between models and elected to use a mono-exponential model instead [41].

#### Phosphagen Anaerobic Contribution: Muscle Estimates

An alternative approach to estimate phosphagen energy release has been to calculate the amount of energy derived from maximal PCr splitting in the contracting muscle. The MM studies in this review that have used a muscle-centric estimation approach [45–47, 49–51, 82, 83, 86, 87, 139, 141–143, 145] have done so by indirectly estimating PCr stores in active muscle, assuming complete depletion of PCr during the early phase of maximal exercise, and then subsequently quantifying the ATP–PCr energy release. This method is based on estimates of total body mass and the assumed maximally active muscle mass, the energy equivalent of PCr concentration stores at rest, the time constant of PCr splitting from the commencement of exercise, and the exercise duration [86]. Estimates in literature of the concentration of PCr stores in muscle range from 17.8 to 37.7 mmol/kg muscle (wet weight) [151, 152], with studies here tending to use 18.0–18.5 mmol/kg [46, 82, 86, 142, 143] or an average value (e.g., 27.75 mmol/kg [83]). Estimates of maximally active muscle mass have mostly used 30% body mass in their calculations [45, 82, 86, 142, 143], although 25% [46, 47] and 50% [83] body mass have also been used. A small group of studies [49–51] used anthropometric measures (body mass, height, forearm, thigh and calf girths, and thigh and calf skinfolds) to estimate muscle mass [153]. Estimates of the maximal amount of energy derived from PCr splitting have varied from 0.416–0.418 kJ/kg body mass [87, 141] to the more consistently used 0.468 kJ/kg [82, 83, 86, 142, 143]. By using this energy equivalent, and a P/O_2_ ratio of 6.25 (a measure of the amount of PCr that needs to be split to spare a given amount of O_2_ [152]), an energy equivalent can be calculated on the basis of the individual’s mass and assumed active muscle mass. As an example, for a 70-kg individual with 50% maximally active muscle mass, this would equate to ~ 73 kJ [(27.75 × 70 × 0.5)/6.25] × 0.468 = 72.7 kJ [83]. During maximal exercise, the energy release from PCr splitting tends to reach this value asymptotically, with a time constant of 23.4 s [83, 86, 87, 142, 143]. Several studies have estimated phosphagen energy release from PCr in O_2_ equivalents on the basis of values in literature [154, 155], ranging from 16.0 mL O_2_/kg [46] to 37.0 mL O_2_/kg muscle mass [45, 49–51].

Sousa et al. [156] compared the kinetics of maximal PCr splitting in the contracting muscle to the fast component of $\dot{V}$O_2_ off-kinetics in recovery during maximal swimming trials and found no significant differences between the two approaches. While both methods have been used to quantify the ATP–PCr energy release during maximal exercise, caution is warranted owing to the limitations and potential for error associated with these indirect, noninvasive techniques.

#### Glycolytic Anaerobic Contribution: Blood Lactate

The glycolytic (or anaerobic lactic) energy contribution has consistently been estimated from net lactate accumulation, taken as the peak blood lactate measured during recovery minus the blood lactate at rest. The energy equivalent of blood lactate accumulation appears to be in the range of 2.7–3.3 mL O_2_/kg of body mass for each 1-mmol increase in blood lactate, with 3.0 mL O_2_/kg given for running and 2.7 mL O_2_/kg for swimming [154, 157]. The energy equivalent of O_2_ (20.9–21.1 kJ/L) is subsequently required to convert to an energy value. Most MM studies [39–41, 45, 49–51, 63, 64, 66, 69–71, 84, 86, 88, 140, 141, 147] used 3.0 mL O_2_/kg as an energy equivalent for the measured increase in blood lactate in their calculations, with slightly higher values of 3.1 mL O_2_/kg [139] and 3.3 mL O_2_/kg [46] also reported. Four swimming studies [82, 83, 142, 143] used 2.7 mL O_2_/kg as previously recommended [154, 157] but two studies did not [86, 139]. Several studies reported and calculated an energy value from the increase in lactate using 0.0627 kJ/mmol/kg [81, 87] or 0.0689 kJ/mmol/kg [158] as opposed to the O_2_ equivalent (2.7–3.3 mL O_2_/kg) reported by most others.

### Theoretical Models

Several studies within this review have reported energy system contributions for sprinting and running performances on the basis of a theoretical mathematical modeling approach to the problem (theoretical model [TM]) [35–38, 74, 75, 159]. These studies were all published in the early period of this review (i.e., 1985–2006) and have an interest in prediction methods that relate distance and running time [36, 38]. These methods are based on the energetics of running and converting chemical energy to external mechanical work and thermal energy [36] rather than alternative earlier attempts based on Newton’s second law of motion (i.e., rate of change of momentum is equal to the sum of applied forces) to derive a relationship between running speed, distance traveled, and time from rest (e.g., [160, 161]).

The relationship between a runner’s power output and the total duration of a race can be described by the hyperbolic function based on the capacity of the anaerobic metabolism (J/kg) and the rate of energy release from aerobic metabolism [38]. The energetics model is based on similar theoretical concepts to many of the studies described under MM in this review and draws on data published in literature to estimate various capacities, rates and constants related to energy release, and/or calculations derived from direct measures for some variables (e.g., maximal aerobic power, blood lactate, speed, race time, body mass, air resistance).

Within the TM group, some studies focused only on a single short maximal effort such as a 100-m sprint [35, 74]) or 600-m middle-distance run [75]) while others modeled performance across a range of distances (100–10,000 m [36]; 800–5000 m [37]; 800–10,000 m [159]; 60–42,195 m [38]). Predictions were made on the basis of known Olympic [36], World Championship [35, 74], and World Record [37, 38, 159] data, as well as assessments and comparisons between actual and predicted running performance for elite junior [75], intermediate [37], and senior [159] athletes, and an elite hypothetical athlete [37].

The empirical model described by Peronnet and Thibault [38] attempted to predict the average power output (W/kg) sustained over any time period on the basis of the capacity of anaerobic metabolism (J/kg), the maximal aerobic power (above basal metabolic rate) (W/kg), and the reduction in peak aerobic power with the natural logarithm of race duration. Several time constants were included to account for the kinetics of aerobic and anaerobic metabolism from the beginning of exercise. In terms of anaerobic contributions, some authors have utilized a two-component model (i.e., phosphagen and glycolytic) [36, 37], while others have expanded this to a three-component model by considering the phosphagen component as the kinetics of ATP and PCr separately.

## Results

### Key Authors

From the 102 included studies, there were 98 unique sets of authors, with only two single authored studies ([36, 45]. Key authors (plus co-authors) included seven studies by Gastin [48, 56, 65, 90, 107, 121, 136], six by Bishop [68, 98–100, 117, 134] and Dawson [49–51, 98, 99, 109], five by Bertuzzi [63, 68, 70, 81, 134], and four by Beneke [39, 66, 88, 144] and Franchini [63, 81, 84, 85].

## Measurement Approaches

Many studies reported data over multiple exercise durations and/or used a second measurement approach for comparative purposes [49–51, 69, 70]. As a result, there were 311 individual data points available for analysis. Each unique trial (i.e., data point) represented the mean energy system contributions from the participants of a given study for the duration, distance, or event completed. The measurement approaches reported were the OD (66 studies, 192 trials; 62% of the data; Table 2), followed by MM (33 studies; 81 trials; 26%; Table 3) and TM (7 studies; 37 trials; 12%; Table 4).

**Table 2:** Estimates of relative anaerobic and aerobic energy system contribution during single bouts of maximal high intensity exercise

Study	Exercise	Participants	*N*	Sex	Age	Duration (s)	Anaerobic (%)	Aerobic (%)
McGawley and Bishop (2015) [117]	Cycle	Soccer players	8	F	26.7	6.0	90.2	9.8
Serresse et al. (1988) [53]	Cycle	CC skiers, biathletes, speed-skaters	23	M	20.6	10.0	97.0	3.0
						30.0	72.0	28.0
						90.0	54.0	46.0
Duffield et al. (2004) [49]	Run	Club-, state-, and national-level track athletes	9	M	25.0	11.5	91.1	8.9
						11.5	79.4	20.6
						13.1	89.1	10.9
						13.1	75.0	25.0
Gastin and Lawson (1994) [107]	Cycle	–	8	M	22.0	15.0	81.4	18.6
						30.0	70.7	29.3
						45.0	61.2	38.8
						60.0	54.1	45.9
						75.0	47.5	52.5
						90.0	42.3	57.7
Hermansen and Medbø (1984) [2]	Run	Well trained	1	–	–	15.0	78.0	22.0
						30.0	71.0	29.0
						60.0	54.0	46.0
						120.0	41.0	59.0
						240.0	25.0	75.0
Spencer and Gastin (2001) [48]	Run	Runners	3	M	19.0	22.3	71.0	29.0
						49.3	57.0	43.0
						113.0	34.0	66.0
						235.0	16.0	84.0
Calbet et al. (1997) [101]	Cycle	Untrained	19	M	23.0	30.0	77.1	22.9
						45.0	69.1	30.9
						147.9	41.5	58.5
Kavanagh and Jacobs (1988) [113]	Cycle	Untrained	5	M	–	30.0	81.5	18.5
O’Brien et al. (1997) [65]	Cycle	–	9	M	20.0	30.0	70.0	30.0
						30.0	67.0	33.0
						60.0	54.0	46.0
						60.0	52.0	48.0
Withers et al. (1991) [54]	Cycle	Cyclists and fit noncyclist	6	M	25.0	30.0	72.0	28.0
						60.0	51.0	49.0
						90.0	39.0	61.0
Granier et al. (1995) [108]	Cycle	Sprinters	7	M	20.7	30.0	81.0	19.0
						30.0	71.0	29.0
						30.0	70.0	30.0
						30.0	55.0	46.0
						30.0	72.0	28.0
						30.0	55.0	45.0
Nummela et al. (1996) [125]	Cycle	Physically active	13	M	24.9	30.0	80.7	19.3
Peyrebrune et al. (2014) [60]	Swim (pool)	Elite swimmers	8	M	20.3	30.0	75.0	25.0
Medbø and Tabata (1989) [52]	Cycle	–	17	M	25.0	34.0	70.0	30.0
						75.0	53.0	47.0
						156.0	35.0	65.0
Medbo and Tabata (1993) [118]	Cycle	Healthy, active	16	M	25.0	34.4	69.0	31.0
						70.0	54.0	46.0
						154.0	34.8	65.2
Ogura et al. (2006) [72]	Cycle	University level	7	M	20.0	40.0	69.3	30.7
						40.0	75.2	24.8
						40.0	75.8	24.2
Nakagaki et al. (2008) [124]	Kayak (ergo)	Kayak paddlers	8	M	20.0	40.0	71.0	29.0
						120.0	43.0	57.0
						240.0	26.0	74.0
Foster et al. (2004) [106]	Cycle	Regional level	14	Mixed	29.2	40.3	75.0	25.0
						87.4	54.7	45.3
						133.8	47.3	52.7
						296.0	29.8	70.2
Withers et al. (1993) [137]	Cycle	Cyclists	12	–	25.1	45.0	60.0	40.0
						60.0	53.0	47.0
						75.0	46.0	54.0
						90.0	40.0	60.0
Nummela and Rusko (1995) [126]	Run	Athletes	6	M	26.1	49.4	54.4	45.6
						49.5	62.9	37.1
Ho et al. (2013) [111]	Kayak (ergo)	Elite Japanese dragonboat paddlers	11	M	21.4	50.0	47.9	52.1
						110.0	32.5	67.5
Spencer et al. (1996) [136]	Run	Runners	4	M	30.0	52.0	54.0	46.0
						118.0	31.0	69.0
						242.0	17.0	83.0
Duffield et al. (2005) [51]	Run	Club- to national-level athletes	11	M	21.8	52.2	64.8	35.2
						52.2	58.7	41.3
						60.2	63.0	37.0
						60.2	55.5	44.5
Zouhal et al. (2010) [138]	Run	National level	6	M	24.2	55.0	62.5	37.4
						61.7	57.0	43.0
Morton and Gastin (1997) [121]	Swim (bench)	Untrained	7	M	21.0	60.0	39.6	60.4
						60.0	44.2	55.8
Cruz et al. (2016) [105]	Cycle	Recreationally active	15	M	–	60.0	39.8	60.2
Gastin et al. (1995) [90]	Cycle	–	9	M	27.0	62.0	49.0	51.0
						90.0	43.0	57.0
						94.0	41.0	59.0
						186.0	24.0	76.0
						208.0	26.0	74.0
Olesen et al. (1994) [127]	Run	Runners	6	–	28.0	62.0	63.2	36.8
						64.0	55.9	44.1
						139.0	40.3	59.7
						146.0	28.2	71.8
						148.0	42.8	57.2
						148.0	31.6	68.4
Craig et al. (1995) [104]	Cycle	Cyclists	6	M	19.5	70.0	49.8	50.2
						70.0	44.7	55.3
						120.0	36.5	63.5
						120.0	32.7	67.3
						140.0	25.7	74.3
						140.0	29.1	70.9
						300.0	14.3	85.8
						300.0	15.1	84.9
Gastin and Lawson (1994) [56]	Cycle	–	8	–	20.0	90.0	44.0	56.0
						90.0	47.0	53.0
						90.0	42.0	58.0
Kon et al. (2019) [114]	Cycle	College level	9	M	20.7	90.0	46.0	54.0
						90.0	47.9	52.1
						90.0	45.0	55.0
						90.0	44.6	55.4
Muniz-Pumares et al. (2017) [123]	Cycle	Cyclists and triathletes	20	M	41.0	91.0	45.1	54.9
						123.0	37.8	62.2
						173.0	29.9	70.1
						267.0	19.1	80.9
Macdermid et al. (2019) [116]	Kayak (field)	New Zealand slalom development team	8	–	–	91.6	32.0	68.0
Simmonds et al. (2010) [135]	Cycle	Highly trained	8	M	26.0	93.5	36.1	63.9
Zouhal et al. (2012) [55]	Kayak (field)	Elite national level	7	M	21.9	108.0	21.7	78.3
						224.0	13.4	86.6
Craig and Morgan (1998) [102]	Run	Middle distance runners	14	M	24.7	115.8	38.8	61.2
						146.9	26.9	73.1
Hettinga et al. (2007) [110]	Cycle	Cyclists	9	M	26.4	116.4	49.4	50.6
						117.1	49.2	50.8
						117.9	48.7	51.3
de Poli et al. (2016) [69]	Run	Recreational	18	M	29.0	118.8	43.6	56.4
Bishop et al. (2001) [98]	Kayak (ergo)	Kayakers	8	Mixed	21.0	120.0	34.3	65.7
						120.0	34.2	65.8
						120.0	31.9	68.1
Bishop et al. (2003) [100]	Kayak (ergo)	State level	7	M	24.0	120.0	40.9	59.1
						120.0	39.7	60.3
Bishop et al. (2002) [99]	Kayak (ergo)	Kayak paddlers	8	M	22.0	120.0	37.7	62.3
						120.0	39.1	60.9
Minahan and Wood (2008) [120]	Cycle	Untrained	8	M	23.0	121.8	32.9	67.1
						129.0	32.0	68.0
						138.0	30.9	69.1
Bickham and Le Rossignol (2004) [97]	Run	Runners	7	M	27.0	135.7	32.7	67.3
						160.3	29.3	70.7
Arezzolo et al. (2020) [95]	Cycle	Cyclists	9	–	35.1	156.0	36.6	63.4
						156.0	33.3	66.7
						164.0	35.4	64.6
						164.0	33.1	66.9
						165.0	35.0	65.0
						165.0	31.4	68.6
						167.0	35.2	64.8
						167.0	32.3	67.7
						177.0	34.3	65.7
						177.0	30.6	69.4
						185.0	33.5	66.5
						185.0	29.3	70.7
Muniz-Pumares et al. (2017) [122]	Cycle	Cyclists and triathletes	21	M	40.0	164.0	29.7	70.3
						180.0	23.7	76.3
Minahan et al. (2007) [119]	Cycle	Recreationally active	7	F	23.0	167.0	31.0	69.0
						171.0	30.0	70.0
						175.0	30.0	70.0
Ramsbottom et al. (1994) [130]	Run	Recreational	12	Mixed	25.8	171.7	30.7	69.3
						181.8	29.3	70.7
						181.8	29.3	70.7
Losnegard et al. (2012) [115]	Ski (treadmill)	Elite senior	12	M	24.0	171.8	26.6	73.4
						172.1	26.2	73.8
Green et al. (1996) [109]	Cycle	Cyclists	10	M	26.0	173.0	24.4	75.6
Ramsbottom et al. (1997) [131]	Run	–	17	F	23.8	173.2	32.4	67.8
						177.6	29.9	70.1
Pouilly and Busso (2008) [128]	Cycle	Active, not highly trained	12	M	22.7	176.0	20.5	79.5
Bangsbo et al. (1993) [96]	Cycle	Cyclists	3	M	23.0	178.8	26.1	73.9
						180.6	21.9	78.1
						205.2	34.5	65.5
						243.0	31.0	69.0
						360.0	33.6	66.4
Lima-Silva et al. (2013) [68]	Cycle	Physically active	6	M	29.7	180.0	33.8	66.2
						222.0	33.7	66.3
						264.0	33.4	66.6
Kalva-Filho et al. (2017) [112]	Swim (pool)	Swimmers	9	Mixed	18.0	180.0	19.7	80.3
Faina et al. (1997) [62]	Cycle	Cyclists	8	M	24.0	225.0	16.4	83.6
						302.0	16.8	83.2
						356.0	11.5	88.5
Andersson et al. (2016) [94]	Ski (treadmill)	Skiers	10	M	24.6	228.0	20.0	80.0
						228.0	19.0	81.0
						230.0	17.0	83.0
						231.0	17.0	83.0
Andersson et al. (2017) [93]	Ski (treadmill)	Skiers	11	M	24.3	232.0	18.0	82.0
Riojas et al. (2020) [132]	Cycle	Recreational	20	Mixed	21.0	246.0	32.3	67.7
						246.0	34.7	65.3
						490.0	12.0	88.0
						490.0	20.4	79.6
Aisbett et al. (2009) [91]	Cycle	Cyclists	26	M	29.0	300.0	16.6	83.4
						300.0	16.3	83.7
						300.0	15.6	84.4
Palmer et al. (2009) [67]	Cycle	National standard	8	–	30.0	338.0	16.4	83.6
Craig et al. (1993) [103]	Cycle	Track and sprint cyclists	18	M	20.1	339.7	16.0	84.0
Aisbett et al. (2003) [92]	Cycle	–	6	M	25.2	360.0	12.1	87.9
						360.0	11.4	88.6
						360.0	12.0	88.0
Russell et al. (1998) [133]	Row (ergo)	Junior rowing crew	19	M	18.0	403.0	16.0	84.0
Santos Rde et al. (2013) [134]	Cycle	Cyclists	8	M	32.6	419.1	26.2	73.8
Pripstein et al. (1999) [129]	Row	Competitive rowers	16	F	21.0	450.0	12.3	87.7
Duffield et al. (2005) [50]	Run	Trained	8	M	26.0	577.7	7.0	93.0
						577.7	14.0	86.0
						695.0	8.0	92.0
						695.0	6.0	94.0

Assessment method: oxygen deficit (*n* = 66 studies)

**Table 3:** Estimates of relative anaerobic and aerobic energy system contribution during single bouts of maximal high intensity exercise

Study	Exercise	Participants	*N*	Sex	Age	Duration (s)	Anaerobic (%)	Aerobic (%)
Sousa et al. (2017) [140]	Run	Active	12	M	21.0	10.0	90.3^c^ (52.7, 37.6)	9.7
						20.0	85.3^c^ (56.0, 29.3)	14.7
						30.0	79.4^c^ (53.9, 25.5)	20.6
Bogdanis et al. (1998) [34]	Cycle	University students	8	M	26.0	10.0	87.0^a^	13.0
						20.0	73.0^a^	27.0
Locatelli and Arsac (1995) [47]	Run	Runners	4	M	–	10.6	97.7^b^	2.3
						11.8	96.8^b^	3.2
Duffield et al. (2004) [49]	Run	Club-, state-, and national-level track athletes	8	M	22.3	23.8	79.3^b^	20.7
						23.8	71.6^b^	28.4
						26.8	78.0^b^	22.0
						26.8	66.8^b^	33.2
Bernardi et al. (2007) [146]	Arm crank ergo	Grinders and mastmen	6	M	28.0	27.0	85.0^c^ (56.0, 29.0)	15.0
Smith and Hill (1991) [145]	Cycle	–	6	M	–	30.0	84.0^b^ (56.0, 28.0)	16.0
Beneke et al. (2002) [39]	Cycle	Rugby players	11	M	21.6	30.0	81.4^c^ (50.3, 31.1)	18.6
Ozkaya et al. (2014) [64]	Elliptical	Mixed sport backgrounds	12	M	20.3	30.0	88.8^c^ (45.0, 43.8)	11.2
						30.0	84.3^c^ (45.3, 39.1)	15.7
Micklewright et al. (2006) [144]	Cycle	Physically active	15	M	24.0	30.0	78.1^c^ (40.9, 37.2)	21.9
						30.0	77.5^c^ (40.0, 37.5)	22.5
Leithauser et al. (2016) [88]	Cycle	No details	10	M	26.6	30.0	86.0^c^ (59.0, 27.0)	14.0
						30.0	87.0^c^ (61.0, 26.0)	13.0
La Monica et al. (2020) [41]	Cycle	Recreationally active	11	M	22.8	30.0	90.3^c^ (46.9, 43.4)	9.7
						30.0	89.9^c^ (45.1, 44.8)	10.1
						30.0	90.2^c^ (46.8, 43.4)	9.8
						30.0	89.8^c^ (47.6, 42.3)	10.2
						30.0	89.2^c^ (46.1, 43.1)	10.8
Doria et al. (2020) [71]	Cycle	Mountain climbers	7	M	39.4	30.0	80.1^c^ (48.3, 31.8)	19.9
						30.0	81.7^c^ (43.1, 38.6)	18.3
Lovell et al. (2013) [147]	Arm crank ergo	Physically active	14	M	24.0	30.0	88.6^c^ (60.3, 28.3)	11.4
Franchini et al. (2016) [84]	Arm crank ergo	Well trained	14	M	21.0	30.0	79.0^c^ (46.0, 33.0)	21.0
Julio et al. (2019) [85]	Cycle	State or national level	11	M	18.0	30.0	77.0^c^ (45.0, 32.0)	23.0
						30.0	79.0^c^ (50.0, 29.0)	21.0
Figueiredo et al. (2011) [83]	Swim (pool)	International-level swimmers	10	M	21.6	32.2	55.4^b^ (14.1, 41.3)	44.6
						141.3	34.1^b^ (13.6, 20.4)	65.9
Bottollier et al. (2020) [141]	Ski	Alpine ski racers	8	Mixed	18.2	43.9	65.7^b^ (36.3, 29.4)	34.3
						52.9	60.1^b^ (34.6, 25.5)	39.9
						53.2	56.1^b^ (26.5, 29.6)	43.9
						78.3	51.5^b^ (29.9, 21.6)	48.5
Lacour et al. (1990) [46]	Run	Top-level runners	17	Mixed	25.4	47.5	71.8^b^	28.2
						108.4	50.3^b^	49.7
Hill (1999) [45]	Run	Runners	6	M	–	49.3	63.0^b^	37.0
						61.2	62.0^b^	38.0
						120.2	39.0^b^	61.0
						145.7	33.0^b^	67.0
						245.8	20.0^b^	80.0
						308.5	17.0^b^	83.0
Wittekind and Beneke (2011) [66]	Cycle	Cyclists or triathletes	11	M	31.0	60.0	63.0^c^ (38.0, 25.0)	37.0
						60.0	62.0^c^ (36.0, 27.0)	38.0
						60.0	60.0^c^ (34.0, 26.0)	40.0
Bertuzzi et al. (2007) [81]	Climb	Climbers	6	–	20.1	73.8	58.4^c^ (8.4, 50.1)	41.6
						80.8	51.5^c^ (12.3, 39.3)	48.5
						82.3	56.1^c^ (13.8, 42.5)	43.9
						83.9	57.9^c^ (16.4, 41.5)	42.1
Zamparo et al. (2006) [87]	Kayak (field)	Italian national whitewater team	8	–	24.8	85.7	54.8^b^ ((29.9, 24.9)	45.2
						88.1	53.0^b^ (33.9, 19.0)	47.0
de Poli et al. (2016) [69]	Run	Recreational	18	M	29.0	118.8	43.6^c^ (25.0, 18.6)	56.4
Sousa et al. (2014) [86]	Swim (pool)	Swimmers	12	M	18.2	122.6	41.0^b^ (20.0, 21.0)	59.0
						194.2	26.0^b^ (12.0, 14.0)	74.0
						344.1	17.0^b^ (7.0, 10.0)	83.0
Duffield et al. (2005) [51]	Run	Club- to national-level athletes	9	M	19.8	126.0	36.6^b^	63.4
						126.0	39.7^b^	60.3
						151.5	31.4^b^	68.6
						151.5	29.9^b^	70.1
de Poli et al. (2019) [70]	Run	Recreationally active	14	M	24.0	160.8	35.0^c^ (19.9, 15.1)	65.0
Brisola et al. (2015) [40]	Run	Healthy and moderately active men	15	M	23.4	163.0	33.4^c^ (19.5, 14.2)	66.6
Ferreira et al. (2015) [143]	Swim (pool)	Swimmers	14	M	35.6	171.2	23.9^b^ (12.0, 11.9)	76.1
						174.8	26.0^b^ (13.1, 12.8)	74.0
						177.5	28.4^b^ (14.8, 13.6)	71.6
						193.4	21.6^b^ (9.3, 12.3)	78.4
						197.4	20.6^b^ (8.0, 12.7)	79.4
						205.2	23.1^b^ (9.9, 13.2)	76.9
Correia et al. (2019) [142]	Swim (pool)	Swimmers	14	Mixed	23.0	171.7	12.2^b^ (6.0, 6.2)	87.8
Ferreira et al. (2016) [82]	Swim (pool)	Participated in national swimming events	11	F	34.7	193.4	21.6^b^ (9.3, 12.3)	78.4
						197.4	20.6^b^ (8.0, 12.7)	79.4
						205.2	22.3^b^ (9.6, 12.8)	77.7
Laffite et al. (2004) [139]	Swim (pool)	Elite	7	M	19.1	255.8	18.9^b^	81.1
Duffield et al. (2005) [50]	Run	Trained	10	M	25.0	263.0	19.0^b^	81.0
						263.0	23.0^b^	77.0
						316.7	18.0^b^	82.0
						316.7	14.0^b^	86.0
de Campos Mello et al. (2009) [63]	Row (ergo with slide)	Competitive rowers	8	M	23.8	398.0	16.0^c^ (7.0, 9.0)	84.0
						402.0	16.0^c^ (7.0, 9.0)	84.0
						515.0	13.0^c^ (6.0, 7.0)	87.0

Assessment method: mixed methods (*n* = 33 studies)

Method used to calculate adenosine triphosphate and phosphocreatine (ATP–PCr) energy release and contribution: ^a^Muscle measures; ^b^Muscle estimates; ^c^Fast-component of post exercise oxygen consumption

**Table 4:** Estimates of relative anaerobic and aerobic energy system contribution during single bouts of maximal high intensity exercise

Study	Exercise	Participants	*N*	Sex	Age	Duration (s)	Anaerobic (%)	Aerobic (%)
Péronnet and Thibault (1989) [38]	Run	Runners		M	–	6.4	94.7	5.3
						9.8	92.4	7.6
						19.8	85.9	14.1
						44.1	69.9	30.1
						101.7	43.0	57.0
						132.2	35.2	64.8
						209.5	23.9	76.1
						226.3	22.3	77.7
						290.8	18.1	81.9
						452.1	12.0	88.0
						778.4	6.3	93.7
						1633.8	2.5	97.5
Arsac and Locatelli (2002) [35]	Run	Sprinters		M	–	9.2	96.0	4.0
						11.5	94.0	6.0
						12.1	95.0	5.0
						13.2	94.0	6.0
Ward-Smith and Radford (2000) [74]	Run	Elite sprinters		M	–	10.2	92.5	7.5
						11.0	90.5	9.5
Ward-Smith (1985) [36]	Run	Runners		–	–	10.2	93.0	7.0
						20.4	86.0	14.0
						44.9	72.0	28.0
						105.5	48.0	52.0
						218.2	28.0	72.0
						816.5	8.0	92.0
						1686.8	4.0	96.0
Billat et al. (2004) [75]	Run	Middle distance	8	M	18.0	86.5	38.2	61.8
						101.6	41.5	58.5
Di Prampero et al. (1993) [37]	Run	Runners	16	Mixed	18.3	102.0	38.0	62.0
						132.0	31.0	69.0
						209.0	22.0	78.0
						452.0	11.0	89.0
						778.0	7.0	93.0
Busso and Chatagnon (2006) [159]	Run	2004 World record		–	–	206.0	24.4	75.6
						227.0	22.3	77.7
						441.0	12.4	87.6
						486.0	11.4	88.6
						757.0	7.5	92.5
						841.0	6.9	93.1

Assessment method: theoretical models (*n* = 7 studies)

### Sample and Participants

Sample size ranged from 1 to 32 participants. The most frequent sample size was 8 participants (16% of all reported trials). Participant age ranged from 18–41 years, with a mean age of 24.4 ± 4.9 years. Participants were predominantly male (78%), with a small proportion of female (12%) or mixed (11%) samples. The training status of participants was described as trained (78%), untrained (20%), or a mix (2%) of trained and untrained participants in the same sample.

### Exercise Mode and Setting

Cycling and running were the most popular exercise modes, accounting for 44% and 36%, respectively. Less frequent modes included swimming (7%), kayaking (5%), skiing (4%), rowing (2%), arm cranking (2%), and climbing (1%). Overall, 69% of trials were undertaken in a laboratory setting.

### Exercise Duration

Exercise duration ranged from 6 to 1686 s. One third of trials were 60 s or less (34%), with a further 20% between 60 and 120 s. Thereafter, the proportion of trials diminished for each subsequent 60 s in duration: trials lasting between 120 and 180 s (19%), 180 and 240 s (10%), 240 and 300 s (6%), 300 and 600 s (8%), and greater than 600 s (3%). The longest duration for a cycling trial was 490 s (range 6.0–490 s), while for running it was 1686 s (range 6.441–1686 s). Theoretical modeling accounted for the seven longest trials (757–1687 s).

### Exercise Pace and Intensity

The exercise pace prescribed was either self-paced (38%), all-out (34%), or constant (28%). A constant pace or intensity was possible during laboratory testing using an exercise ergometer with the selection of a set load or speed. Self-paced included field (actual race, simulated race, or time trial) or laboratory (time trial or exercise to exhaustion) trials. All-out was typical of a Wingate style protocol in the laboratory.

Exercise intensity was only reported in a small proportion (14%) of trials. Where reported, these intensities ranged from 95 to 133% $\dot{V}$O_2_max, with half of these being at 120% $\dot{V}$O_2_max.

### Energy System Contribution

Estimates of relative energy system contribution during single bouts of dynamic maximal high intensity exercise are presented in Tables 2, 3, and 4. Data have been grouped on the basis of measurement approach and are presented first by duration and second by study authors.

An exponential two-phase association model (GraphPad Prism, V10.1, 2023) was used to describe the data, defined as: *Y* = *Y*0 + SpanFast × (1 − exp(− KFast × *X*)) + SpanSlow × (1 − exp(− KSlow*X)), with SpanFast = (Plateau − *Y*0) × PercentFast × 0.01, and SpanSlow = (Plateau − Y0) × (100 − PercentFast) × 0.01.

Model parameters associated with data points up to a duration of 900 s (*n* = 309) were: *Y*0 = 0; Plateau = 100; PercentFast = 71.32; KFast = 0.01345 and KSlow = 0.001662; SpanFast = 71.131896 and SpanSlow = 28.048104.

**Fig. 4:**
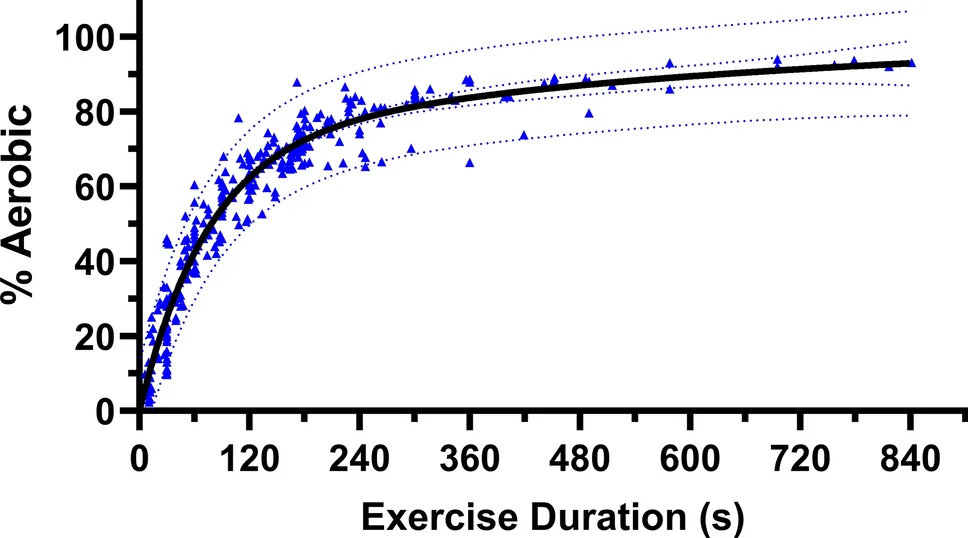
Relative aerobic contribution to the total energy supply during periods of *maximal exercise* (*n* = 309; range 6–841 s). Each data point represents the mean aerobic contribution reported by a study for a duration of maximal exercise. The predicted output of the exponential two-phase association model is displayed (solid black line) along with 95% confidence interval (CI) and 95% prediction error (PE) profile (inner and outer dotted lines, respectively). Goodness of fit at 95% confidence: *R* squared = 0.933. CI ± 0.1 to 6.0%. PE: ± 12.6 to 13.9%

Figure 4 shows data points from each study for the aerobic contribution to the total energy supply for each maximal high intensity exercise bout. The predicted output of the exponential two-phase association model is displayed (solid black line) along with 95% confidence interval (CI) and 95% prediction error (PE) profile (inner and outer dotted lines, respectively). The goodness of fit provided an *R*-squared value of 0.933.

The model output was used to develop estimates of anerobic and aerobic energy system contribution to various durations of maximal exercise (Table 5). A duration of 10 s of maximal exercise was estimated to be 91% anaerobic and 9% aerobic. For 60 s, it was 58% and 42%, respectively. Approximately 75–80 s represented the cross-over point for energy system contribution. On the basis of the modeling, the duration that derived equal contributions from both the anaerobic and aerobic energy systems was 78.6 s (CI ± 1.1 s), with longer durations having an increasingly greater proportion of aerobic energy to the total energy supply.

**Table 5:** Estimates of relative anaerobic and aerobic energy system contribution during selected periods of maximal exercise

Duration of maximal exercise (s)	% Anaerobic	% Aerobic	95% CI
0–5	95	5	0.3
0–10	91	9	0.6
0–15	86	14	0.8
0–20	82	18	0.9
0–30	75	25	1.1
0–45	66	34	1.2
0–60	58	42	1.2
0–75	51	49	1.1
0–90	46	54	1.0
0–120	38	62	1.1
0–180	28	72	1.2
0–240	22	78	1.3
0–300	19	81	1.6
0–600	11	89	2.8
0–900	6	94	6.1

Estimates based on data shown in Fig. 4 and presented in Tables 2, 3, and 4

**Fig. 5:**
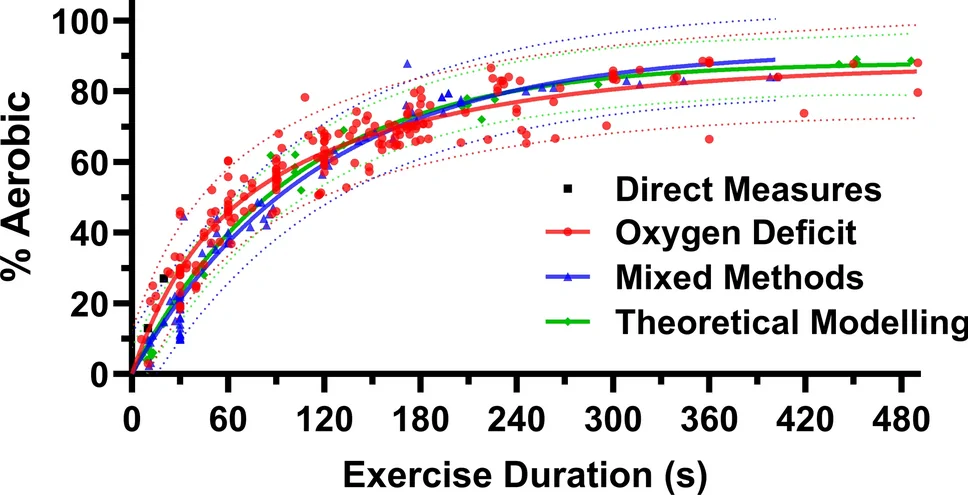
Relative aerobic contribution to the total energy supply for different *methods of measurement*: Direct measures (*n* = 2; range 10–20 s); *oxygen deficit* (*n* = 103; range 6–490 s; *R* squared = 0.897; 95% CI ± 0.1 to 5.2%; PE: ± 12.2 to ± 13.2%); *mixed methods* (*n* = 87; range 10–402 s; *R* squared = 0.956; 95% CI ± 0.1 to 5.3%; PE: ± 11.5 to ± 11.5%); *theoretical modeling* (*n* = 109; range 6–486 s; *R* squared = 0.989; 95% CI ± 0.1 to 6.3%; PE: ± 7.5 to ± 8.7%). Exponential two-phase association model (solid lines) and 95% prediction error (dotted lines). Oxygen deficit curve is significantly different (*p* = 0.001)

**Fig. 6:**
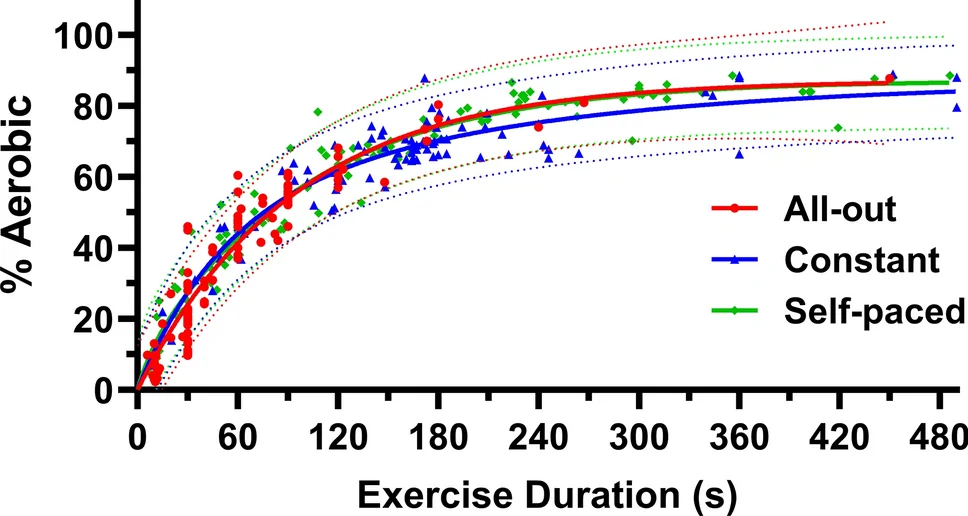
Relative aerobic contribution to the total energy supply during different *pacing strategies* of maximal exercise: *all-out* (*n* = 103; range 6–450 s; *R* squared = 0.888; 95% CI ± 0.1 to 11.9%; PE: ± 14.9 to ± 19.8%); *self-paced* (*n* = 109; range 6–486 s; *R* squared = 0.954; 95% CI ± 0.1 to 3.3%; PE: ± 10.8 to ± 11.2%); *constant pace* (*n* = 87; range 10–490 s; *R* squared = 0.867; 95% CI ± 0.1 to 3.5%; PE: ± 11.9 to ± 13.9%). Exponential two-phase association model (solid lines) and 95% prediction error (dotted lines). Constant pace curve is significantly different (*p* = 0.012)

**Fig. 7:**
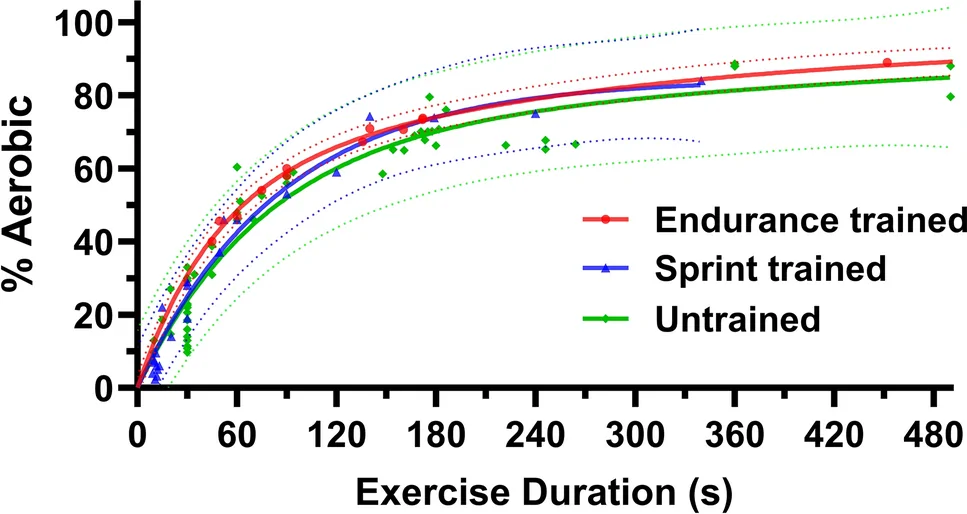
Relative aerobic contribution to the total energy supply for different *training status: endurance trained* (*n* = 15; range 45–1686 s; *R* squared = 0.99; 95% CI ± 0.2 to 2.5%; PE: ± 3.0 to ± 3.9%); *sprint trained* (*n* = 24; range 9–340 s; *R* squared = 0.966; 95% CI ± 0.5 to 10.8%; PE: ± 11.0 to ± 15.4%);. *untrained* (*n* = 57; range 10–490 s; *R* squared = 0.912; 95% CI ± 0.3 to 10.9%; PE: ± 15.7 to ± 19.1%). Exponential two-phase association model (solid lines) and 95% prediction error (dotted lines). Curves are statistically similar (*p* = 0.355)

**Fig. 8:**
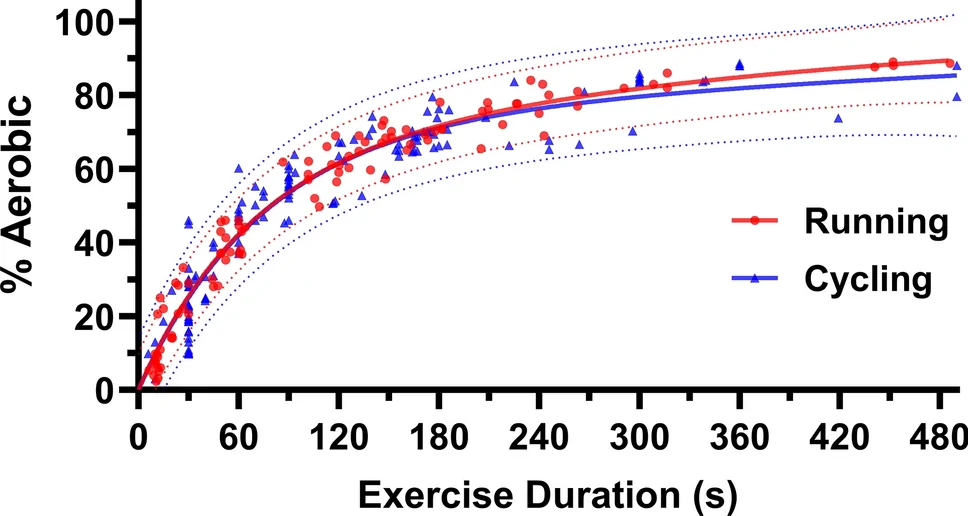
Relative aerobic contribution to the total energy supply during periods of maximal *cycling* (*n* = 134; range 6–490 s; *R* squared = 0.908; 95% CI ± 0.1 to 9.0%; PE: ± 13.8 to ± 16.5%) and maximal *running* (*n* = 101; range 6–486 s; *R* squared = 0.966; 95% CI ± 0.1 to 5.9%; PE: ± 9.7 to ± 11.4%). Exponential two-phase association model (solid lines) and 95% prediction error (dotted lines). Curves are statistically similar (*p* = 0.793)

Several comparisons were visualized to examine different aspects of data categorization and modeling. Figure 5 shows the comparison of measurement methods, Fig. 6 shows a comparison of the pacing strategies, Fig. 7 shows the contrasting of participant training status, and Fig. 8 shows a comparison of the two most frequently performed exercise modes, running and cycling. Data are presented up to 490 s, corresponding to the longest cycling trial, with cycling being the most frequently observed exercise mode. Comparison of the modeled curves revealed no significant differences between running and cycling trials (*p* = 0.793) or between training status groups (*p* = 0.355). In contrast, the measurement method had a small significant effect, with the OD curve differing from both MM and TM (*p* = 0.001). For pacing strategy, constant-intensity exercise differed from both all-out and self-paced conditions (*p* = 0.012).

## Discussion

The primary objective of this review was to systematically evaluate values reported in literature for the relative anaerobic and aerobic contribution to the energy supply during single bouts of maximal exercise. A total of 102 studies reported relevant data, with various methods used to investigate the problem. On the basis of these data, we summarized the energy system contribution (two component model; % anaerobic, % aerobic; Table 5) for varying durations of maximal exercise. The estimates provided are very similar to those published by Gastin in 2001 [14], with differences ranging from 0 to 3% for any given duration. This is partly to be expected given the current systematic review included 28 studies (27%) from the original review. The updated estimates here represent a 2–3% greater aerobic contribution to maximal exercise for durations up to 15 s, similar estimates for a duration of 20 s (i.e., 82% anaerobic, 18% aerobic), and a 1–3% greater anaerobic contribution for durations ranging from 30 to 240 s. Equal contributions from both the anaerobic and aerobic energy systems are at approximately 75–80 s for a single bout of maximal exercise, with a greater contribution from the aerobic system as exercise duration increases. These estimates have a 95% CI of ± < 0.1% to 6.0% and a PE of about 12–14%, meaning the table should be interpreted cautiously. It should also be acknowledged that the summarized table is a result of modeling mean data from all included studies with a range of exercise protocols (mode, duration, pace, intensity, setting) and methods of assessment (OD, MM, TM) used to calculate energy system contribution.

For the modeling of relative aerobic contribution and a comparison between the methods of assessment (see Fig. 5), the nonlinear regression parameters were all similar except for KFast, which was significantly higher for OD, indicating that the extracted data modeled for the OD method had a higher aerobic contribution during the shorter durations of maximal exercise. The reason for this is uncertain and difficult to resolve, as method comparisons are limited. Further, direct measures of anaerobic energy release (i.e., via muscle biopsy, blood flow, and arterial–venous differences) during small muscle group measurements cannot be extrapolated to whole body exercise [43, 44]. Either of the assessment methods could be the more accurate, or the difference could be related to an underestimate of total energy cost in the OD method (subsequently resulting in a greater estimated % aerobic contribution), or an overestimate of anaerobic metabolism in TM and MM (and subsequently lower estimated % aerobic contribution). The differences are however small and most likely of theoretical rather than practical significance. TM had a significantly lower PE than the OD and MM methods, which is to be expected, as unlike the other two methods, TM did not collect experimental data but rather theoretically modeled a small number of data and estimates from literature. The PE was greater for all-out protocols and for cycling (which also had more all-out trials) compared with running. All-out protocols, and to a lesser extent self-paced protocols, allow greater variability in intensity and pacing throughout the trial, which may impact how effectively energy cost can be estimated.

There was considerable variability in the approaches used by the studies reported in this review to measure energy release during maximal exercise and to subsequently estimate the relative energy system contribution from anaerobic (phosphagen and glycolytic) and aerobic pathways to the total energy supply of exercise. As there is no gold standard approach to measure anaerobic energy release during whole-body maximal exercise, the studies reported here typically rely on concurrent (e.g., [43, 49–51, 54, 119, 122]) validity to support their assessment approach to measuring energy release. A range of experimental manipulations, typically used to investigate hypothesized differences or outcomes, may add a further level of ecological support. Examples within the studies reviewed include manipulations related to diet and supplementation [40, 68–70]; duration, distance and intensity [45, 48, 49, 54, 55, 86, 104, 118, 123, 137, 141]; exercise mode [63, 96, 138, 141]; sex [49, 74, 75]; hypoxia [72, 95, 114]; pacing [91, 92, 94, 99, 104, 110, 134]; warm-up [65, 66, 98, 100]; training [41, 95, 97, 120]; and training status [56, 81].

Notwithstanding the differences between, and even within, assessment approaches, the aerobic contribution to the energy supply during maximal exercise of short duration is considerable and appreciably greater than traditionally thought [14, 48]. Oxygen kinetics, measured breath by breath, demonstrate a remarkably fast response of the cardiovascular system to the stimulus of maximal high intensity exercise [14, 56, 90]. Higher intensity exercise and energy demand elicits a faster O_2_ response, observed between sprint and middle distance trials in cycling [162], running [48], and swimming [163]. All-out exercise or time trials characterized by an initial burst of power result in faster O_2_ kinetics compared with constant intensity, square wave exercise [90, 99, 162]. This is further supported by manipulations in pacing strategy with a more rapid increase in $\dot{V}$O_2_ being observed using a fast-start strategy, resulting in an increase in cycle time to exhaustion, likely a result of a sparing of some of the anaerobic capacity during the initial phase of exercise [25, 164]. Interestingly, sprint-trained athletes, despite a lower $\dot{V}$O_2_max, have demonstrated similar or greater aerobic energy release in the first 30 s of a 90-s all-out cycle test than their endurance trained counterparts [56]. Sprint training stimulates muscle adaptations consistent with rapid anaerobic energy release [9, 165] and fast O_2_ kinetics [166] but not the same cardiorespiratory adaptations and maximal aerobic power consistent with sustained endurance training [167].

Understanding the interactions between, and contributions of, energy systems to the overall energy supply during maximal exercise has important practical implications beyond our theoretical knowledge. Data presented here reinforce the concept of a finite anaerobic capacity that limits our ability to exercise at a supramaximal intensity beyond a certain duration. The longer the duration, the lower the average intensity throughout the exercise trial and the lower the contribution of the anaerobic energy system to the total energy delivery. In contrast, the aerobic system operates as a rate or power (i.e., aerobic power with energy delivered per unit of time) and can deliver energy continuously and in an ongoing manner. As a crude illustration, an individual with an OD of 50 mL/kg (O_2_ equivalent) and a $\dot{V}$O_2_max of 50 mL/kg/min may theoretically be able to deliver up to 100 mL/kg O_2_ in a 60-s maximal effort (50% anaerobic: 50% aerobic). In a 120-s effort, this would be 150 mL/kg O_2_ (33%: 67%); in a 180-s effort, this would be 200 mL/kg O_2_ (25%: 75%), and so on, as the OD is a finite capacity while the oxygen uptake provides energy as a rate per minute. However, the anaerobic–aerobic ratios suggested in this simple illustration differ from those presented in Table 5, as in reality, there is a greater anaerobic energy release at the commencement of exercise to meet the energy demand whilst the aerobic system takes time to respond to the exercise requirements.

Faster O_2_ kinetics at the commencement of exercise in some athletes [56] or pacing strategies [25, 164] can result in a sparing of a small portion of the anaerobic capacity and potentially a slightly delayed time to exhaustion or faster time to complete a fixed distance event. The O_2_ response is greater the higher the power output [48, 162, 163, 168] and near maximal values of $\dot{V}$O_2_ can be achieved within 1–2 min of strenuous exercise [107, 162, 169]. Anaerobic energy release at the commencement of exercise is also greater the higher the power output [170]. This strong stimulus and rapid response of the neuromuscular and cardiorespiratory systems to meet the demands of strenuous exercise makes high intensity and supramaximal interval training a powerful and efficient training method to achieve system adaptations for health and performance benefits [171–176].

### Practical Applications

For scientists, these findings highlight the need to design assessment protocols that carefully consider exercise duration, pacing strategy, training status, and the underlying assumptions of the selected measurement approach. Methodological choices—such as constant-load versus all-out testing or the decision to model oxygen cost versus measure it directly—can meaningfully influence estimates of energy system contribution and should therefore be justified and standardized where possible. The review also underscores the value of reporting detailed methodological parameters (e.g., warm-up, intensity, muscle mass assumptions, oxygen equivalents) to enhance comparability across laboratories.

In practical terms for athletes, coaches, and strength and conditioning practitioners, these findings reinforce the importance of aligning training prescription with the specific anaerobic–aerobic demands of the target event. For short-duration, high-power efforts, training should emphasize rapid phosphagen turnover, glycolytic capacity, and tolerance to the immediate metabolic consequences of high-intensity work. As event duration approaches or exceeds the ~ 75–80-s crossover point, programming should increasingly focus on improving aerobic power and accelerating oxygen-uptake kinetics so that athletes can attain high percentages of $\dot{V}$O_2_max early in an effort. Manipulating work-to-rest ratios, incorporating structured fast-start intervals, and using varied-intensity repeats can strategically spare finite anaerobic resources while enhancing the speed and efficiency of aerobic engagement. Athletes can also interpret their training responses more effectively by recognizing that improvements in early aerobic activation or the ability to sustain high oxidative power translate directly to performance gains in efforts lasting longer than 1 min.

### Limitations

This systematic review is limited by the substantial methodological heterogeneity across included studies. Considerable variation existed in exercise protocols (mode, duration, pacing strategy, and intensity), sample characteristics (training status, sex distribution, and athletic background), and the indirect methods used to quantify anaerobic and aerobic energy release. The absence of a gold standard for assessing anaerobic energy release in whole-body maximal exercise means that all included approaches—oxygen deficit, mixed metabolite-based techniques, and theoretical modeling—rely on assumptions that introduce uncertainty. Differences in how studies estimated oxygen demand, mechanical efficiency, PCr availability, lactate accumulation, or fast-component EPOC likely contributed to the variability observed, particularly in short-duration trials where small methodological discrepancies can produce large proportional effects. Furthermore, very few studies employed direct invasive measurements, and the extrapolation of small-muscle-group physiology to whole-body tasks remains problematic.

In addition, the modeling presented here synthesizes mean values across diverse protocols and methods, which increases generalizability but reduces specificity. The derived anaerobic–aerobic contribution estimates therefore represent averaged trends rather than precise predictions for any particular exercise mode, pacing strategy, or athlete population. The prediction error associated with the nonlinear regression model (approximately 12–14%) underscores the need for cautious interpretation of the crossover point, relative contributions at specific durations, and comparisons between measurement methods, exercise mode, and training status. Finally, advancements in measurement technologies or updated methodological standards since the most recent included study (January 2020) were not captured. Future research employing standardized protocols and more direct measures of anaerobic metabolism would help refine these estimates. While ^31^P magnetic resonance spectroscopy shows considerable promise for directly and noninvasively assessing intramuscular energy metabolism and ATP–PCr kinetics during exercise, its application to whole-body maximal efforts remains limited by cost, accessibility, and technical complexity. To date, studies using this technique have focused on localized protocols such as single-leg knee extension [177], plantar flexion [178], or supine cycling [179], which restricts extrapolation to dynamic, multi-joint exercise.

## Conclusions

This systematic review provides a comprehensive update on the relative contributions of anaerobic and aerobic energy systems to the energy supply during single bouts of maximal exercise. The analysis of 102 studies has refined our understanding of the dynamic interplay between these systems across different exercise durations. Our findings emphasize that all energy systems are influenced by exercise duration, although the summarized data should be interpreted cautiously owing to variation in exercise protocols and measurement techniques between studies. More precise or new approaches to the problem may yield further insights into the relative contributions of the phosphagen, glycolytic, and oxidative energy systems to maximal exercise in the future. The modeled data generally corroborate previous summary estimates [14], with minor variations related to a slightly greater aerobic contribution in very-short-duration exercise (< 20 s) and a slightly greater anaerobic contribution as the duration extends from 30 s onward. The outcomes of this review have practical applications for athletes and coaches who aim to optimize performance by manipulating high-intensity exercise prescription factors such as interval durations, work-to-rest ratios, and pacing strategies. Understanding the finite nature of anaerobic capacity and the sustainable power of aerobic energy delivery, combined with profiling each athlete’s unique physiology, can guide training and competition strategies to maximize performance.

## Supplementary Information

Below is the link to the electronic supplementary material.

Supplementary file1 (GIF 1908 KB)

Supplementary file2 (GIF 2262 KB)

Supplementary file3 (GIF 2495 KB)

## Declarations

### Data Availability

Not applicable. No primary data were generated for this review.
