# Supplementary figures and images for: Anaerobic and Aerobic Energy System Contribution During Maximal Exercise: A Systematic Review
Source: Sports Med. 2026 Apr 11;56(7):1723–47. doi: 10.1007/s40279-026-02414-7 (PMC13388521; doi:10.1007/s40279-026-02414-7)

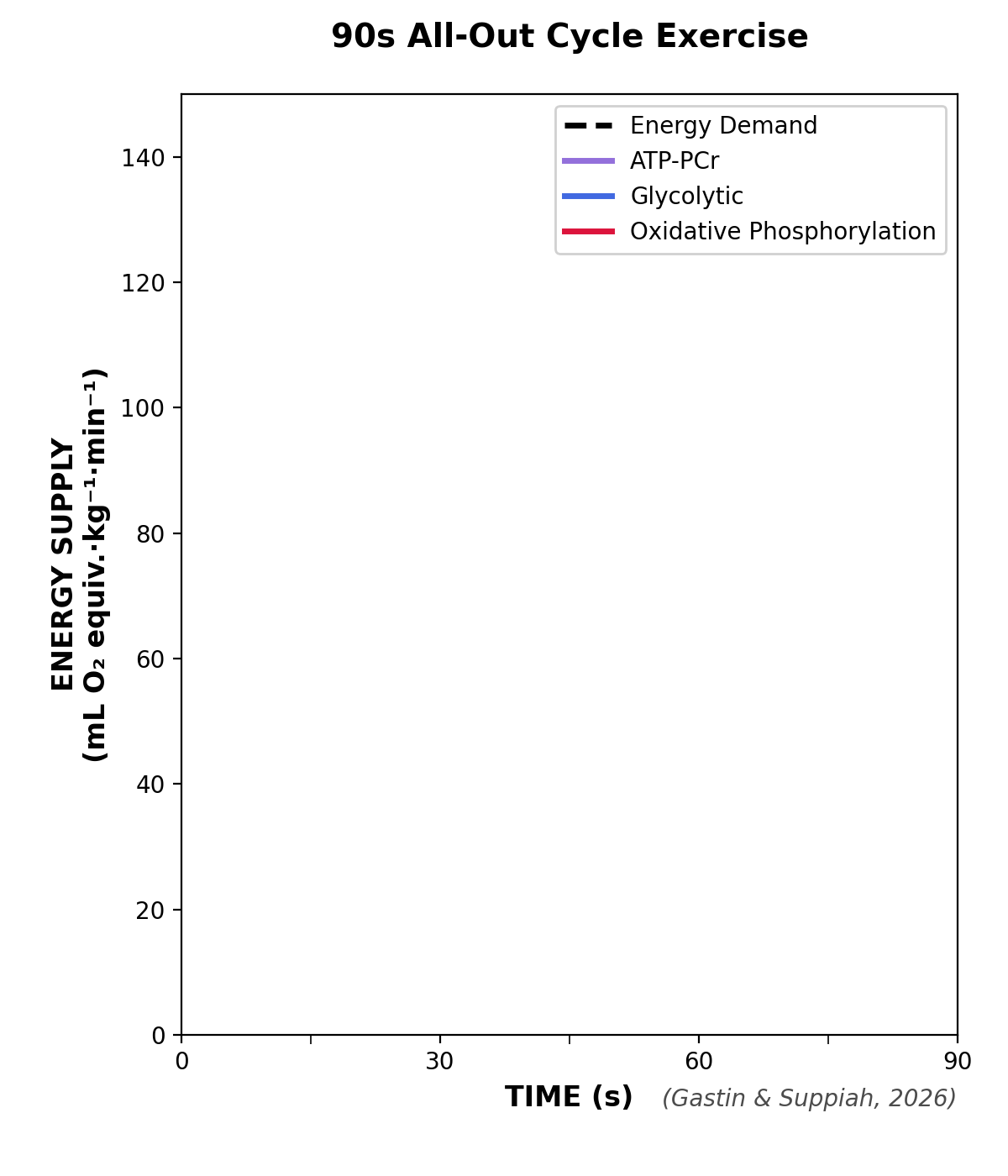

Supplement: Supplementary file 1 — Supplementary file1 (GIF 1908 KB) [file 40279_2026_2414_MOESM1_ESM.gif]

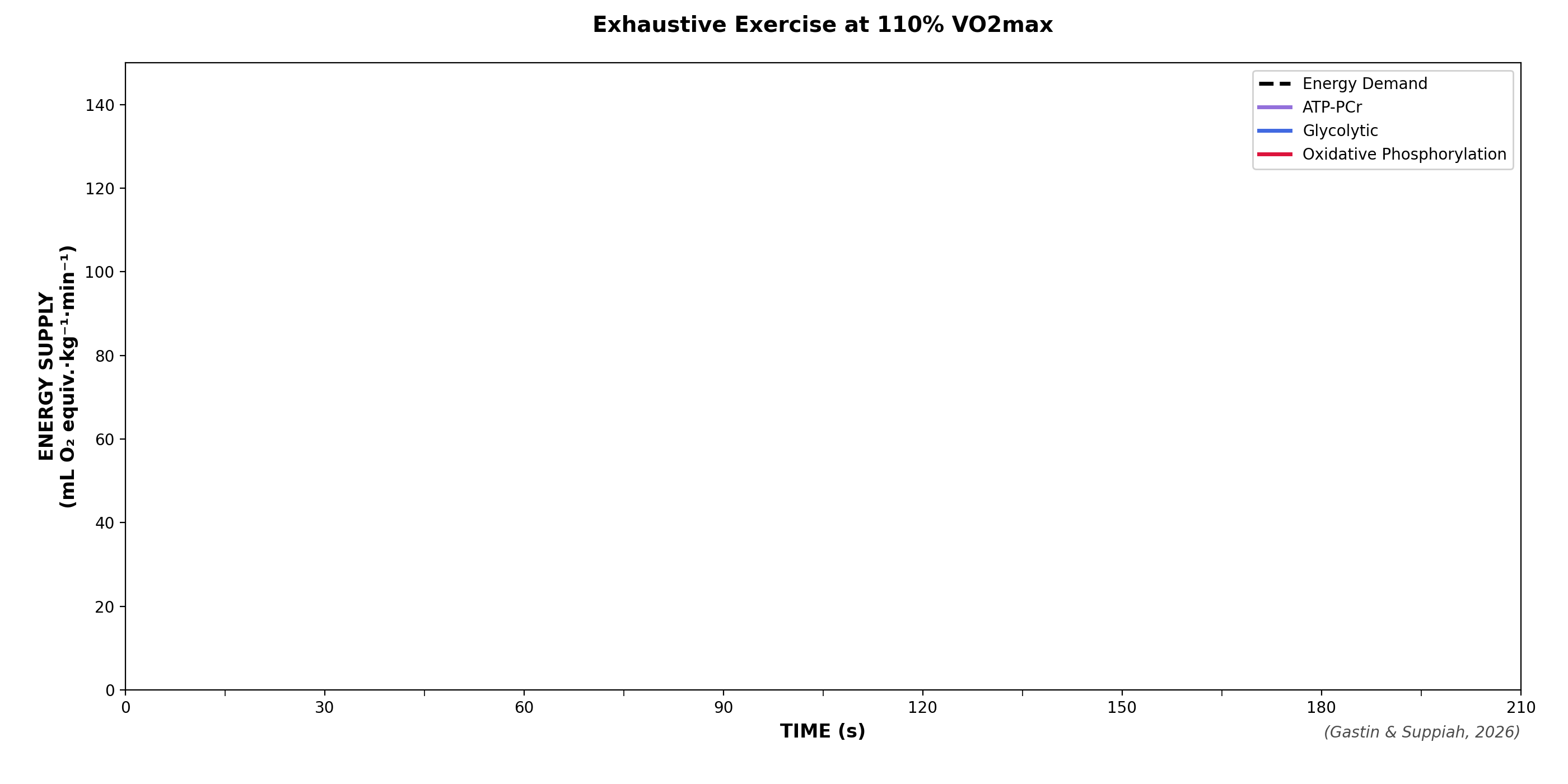

Supplement: Supplementary file 2 — Supplementary file2 (GIF 2262 KB) [file 40279_2026_2414_MOESM2_ESM.gif]

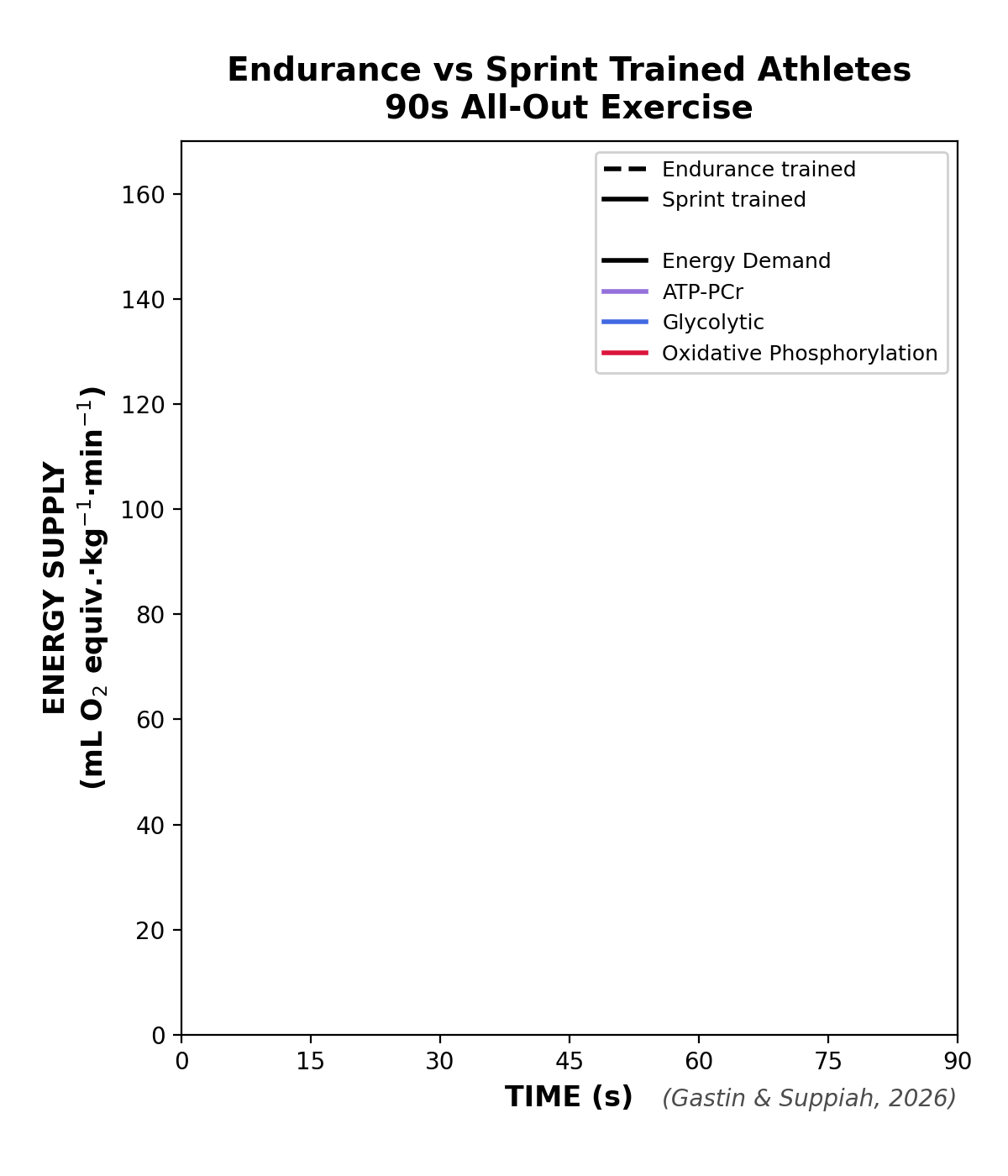

Supplement: Supplementary file 3 — Supplementary file3 (GIF 2495 KB) [file 40279_2026_2414_MOESM3_ESM.gif]
